# Rapid diagnosis of bloodstream infections in the critically ill: Evaluation of the broad-range PCR/ESI-MS technology

**DOI:** 10.1371/journal.pone.0197436

**Published:** 2018-05-15

**Authors:** Martina Tassinari, Silvia Zannoli, Patrizia Farabegoli, Maria Federica Pedna, Anna Pierro, Antonio Mastroianni, Riccardo Fontan, Luciano Luongo, Giuseppe Sarnataro, Elisa Menegatti, Assunta Caruso, Vittorio Sambri

**Affiliations:** 1 Unit of Microbiology, The Great Romagna Area Hub Laboratory, Pievesestina, Italy; 2 Morgagni-Pierantoni Hospital, Forlì, Italy; 3 Santa Maria delle Croci Hospital, Ravenna, Italy; 4 Bufalini Hospital, Cesena, Italy; 5 Umberto I Hospital, Lugo, Italy; 6 Infermi Hospital, Rimini, Italy; 7 DIMES, University of Bologna, Bologna, Italy; Seconda Universita degli Studi di Napoli, ITALY

## Abstract

Bloodstream infection (BSI) and associated sepsis represent a major source of mortality in industrialized countries. Prompt treatment with targeted antibiotics affects both the financial impact and the clinical outcome of BSI: every hour gained in initiating the correct antimicrobial therapy significantly increases the probability of patient survival. However, the current standard-of-care, which depends on blood culture-based diagnosis, are often unable to provide such a fast response. Fast and sensitive molecular techniques for the detection of sepsis-related pathogens from primary blood samples are strongly needed. The aim of this study was to assess the usefulness of the IRIDICA BAC BSI Assay, a PCR/ESI-MS-based technology for the early diagnosis of bloodstream infections from primary blood samples in critical patients. This evaluation has been performed by comparison with the traditional culture-based methods. The study was performed on a total of 300 prospective whole blood specimens obtained from patients suspected of sepsis, admitted to enrolling ER units from The Greater Romagna Area. The overall concordance between the two techniques was of 86%, with a calculated sensitivity of 76% and an assay specificity of 90%. The clinical significance of discrepant results was evaluated reviewing the patients’ clinical records and the results of additional relevant microbiological tests. The data here obtained support the ability of the IRIDICA BAC BSI Assay to identify a broad range of bacteria directly from primary whole blood samples, within eight hours. This might allow a timely administration of a suitable treatment.

## Introduction

Bloodstream infection (BSI) and associated sepsis represent a major issue in industrialized countries [1], with an incidence of three cases per 1,000 individuals in Europe. Sepsis has been shown as the third most frequent cause of death in Germany, and the incidence in France increased by 75% in a ten-year span [2–4].

Prompt treatment with targeted antibiotics affects both the financial impact and the clinical outcome of BSI: every hour gained in initiating the correct antimicrobial therapy significantly increases the probability of patient survival [1, 5–7]. However, the current standard-of-care, which depends on blood culture-based diagnosis, are often unable to provide such a fast response [8–10].

Although many infections can be detected after 24 to 48 hours, it may take up to 5-days incubations to capture the majority of slow-growing bacteria and fungi associated with BSI, and the antibiotic susceptibility determination require an additional 6 to 24 h [10–13].

A targeted therapy, therefore, only follows after several days of empirical treatment with broad-spectrum antibiotics [14]. The empirical use of antibiotics results in a 15–30% rate of inappropriate treatment, which is associated with a 2 to 5- fold increase in the mortality risk of septic patients and a contributing factors in the recent increases in antibiotic-resistant organisms [14–15].

In addition to temporal issues, the diagnostic yeld needs to be considered. Conventional microbiologic methods have a low diagnostic yield, especially in patients treated prior to sampling [6,16], and are not sensitive to unculturable or fastidious organisms, which may be identified with molecular methods [3, 17–18]. However, such methods lack either the sensitivity to identify sepsis-related pathogens directly from the primary sample or a wide enough panel of identification [19–20].

Many culture-negative, molecular-positive detections are likely to be due to culture insensitivity rather than a lack of specificity or clinical relevance of molecular methods [6, 19–21]: many studies reported that these results are frequently confirmed in later cultures; additionally, blood culture is positive in only 50% of cases where BSI is strongly suspected from a clinical point of view [22–24].

Fast and sensitive molecular techniques for the detection of sepsis-related pathogens are urgently needed, especially from primary blood samples [20].

The technology here presented is a universal PCR amplification coupled with mass spectrometry (PCR/ESI-MS) [21]. This method is based on a mismatch and background tolerant PCR reaction, generating amplicons from BSI-related bacteria and Candida species [24–25]. The amplicons are analyzed through mass spectrometrometry; sequence variants are descriminated from a panel of more than 780 species [24, 26–27].

In this study we compared the performance of the IRIDICA BAC BSI Assay, performed on the batch platform IRIDICA System (CE-IVD marked), to that of traditional culture-based methods in a series of clinical whole blood specimens prospectively collected from patients attending selected Units of the Emergency Department and Infectious Diseases of the Great Romagna Health Authority. The aim was to evaluate the usefulness of this method for the early diagnosis of bloodstream infections in patients with suspected sepsis.

## Materials and methods

### Ethics statement

Informed consent was obtained from all patients included in the study. This study was approved by the Clinical Research Ethics Committee of IRST IRCCS-AVR (approval n. 1321/11.06.2015).

### Patients and specimens

300 whole blood samples were prospectively collected between May 1^st^ 2016 and December 31^st^ 2016 from consenting patients who presented to one of the units participating in the study: Emergency Room and Emergency Medicine Units—Bufalini Hospital (Cesena, Italy), Santa Maria delle Croci Hospital (Ravenna, Italy), Infermi Hospital (Rimini, Italy); Infectious Diseases Unit—Morgagni-Pierantoni Hospital (Forlì, Italy), Emergency Medicine Unit—Umberto I Hospital (Lugo, Italy). This study focused on a non-hospitalized population, as these subjects were presumably not under previous antibiotic treatment, which might have affected blood culture results [6,16]; blood withdrawals were performed approximately within 2 hours from admission. The chief selection criterion was a clinical suspicion of sepsis, in accordance with the Third International Consensus Definition for Sepsis and Septic Shock: sepsis is a life-threatening organ disfunction caused by a dysregulated response to infection. Such disfunction is defined by an increase in the Sequential Sepsis-related Organ Failure Assessment (SOFA) score of 2 points or more [28]. Patients were considered eligible for the study if 18 years old or older, were able to personally provide the informed consent, and were having blood cultures drawn as a part of standard clinical care. Specimens were collected in 10 ml EDTA blood tubes following blood draws taken for routine microbiology testing, using the same venipunctures. Then, samples were refrigerated and transported from each hospital to the Unit of Microbiology at the Greater Romagna Area Hub Laboratory in Pievesestina (FC), with an average time of transport of 40 min. The cold chain was maintained and controlled during the transport. Both the PCR/ESI-MS and the microbiology testing, including incubation, were performed at our Laboratory. The IRIDICA BAC BSI Assay and standard-of-care testing were performed blindly to one another’s results. The reports from the microbiology testing were used in this study as the comparative method to evaluate the IRIDICA System.

### Specimen processing with conventional microbiological methods

The blood culture collection policy recommends a minimum of two sets of blood cultures drawn for each patient suspected of sepsis. Processing multiple cultures is useful to increase sensitivity and to discriminate true pathogens from contaminants [3, 29]. Each set consists of an aerobic bottle (BacT/ALERT^®^ FA Plus, bioMérieux, Marcy l’Etoile, France) and an anaerobic lytic bottle (BacT/ALERT^®^ FN Plus, bioMérieux), containing adsorbent polymeric beads for the neutralization of antimicrobial agents, which are inoculated with 10 mL of blood each. When samples arrived at the Laboratory, the two sets of blood cultures were incubated in a BacT/ALERT (bioMérieux) automated instrument for up to 5 days [13]; positive samples were identified through time-of-flight mass spectrometry coupled with matrix-assisted laser desorption/ionization (MALDI-TOF) performed on Vitek MS (bioMérieux) instrument. The susceptibility testing was achieved using the automated Vitek 2 (bioMérieux) system from positive blood culture bottles after performing a Gram stain and a concentration procedure [11–13].

### Specimen processing with IRIDICA–BAC BSI ASSAY

Prior to testing the clinical samples, the instrument functionality was tested through analysis of five reference strains: methicillin-resistant *Staphylococcus aureus*, carbapenem-resistant *Klebsiella pneumoniae*, vancomycin-resistant *Enterocuccous faecium*, vancomycin-resistant *Enterocuccous faecalis*, *Candida albicans* (NATtrol™ ZeptoMetrix Corporation, U.S.A.).

Samples for molecular analysis were processed according to the manufacturer’s instructions using the IRIDICA BAC BSI Assay (List Number 08N22-010, Ibis Biosciences-Abbott Molecular, Des Plaines, IL), leading to microbial identification from whole blood in 6–8 h. Up to 6 specimen may be tested in a single batch. As previously described [3, 30], this process consists in an automated sample lysis using the IRIDICA Bead Beater (BB) instrument, an automated nucleic acid extraction using the IRIDICA Sample Prep (SP) instrument, a PCR amplification using the IRIDICA ThermoCycler (TC) instrument, an automated amplicon purification using the IRIDICA Desalter (DS) instrument, an automated electrospray ionization time-of-flight mass spectrometry using the IRIDICA Mass Spectrometer (MS) instrument and a bioinformatics data analysis with the IRIDICA Analysis Software (AS). Briefly, 5 mL whole blood samples were chemically and mechanically lysed and an extraction control was added to monitor the process. The extraction system uses prefilled disposable cartridges and the eluted fluids were transferred into 16 wells of a custom PCR assay strip prefilled with PCR master mix and 18 primer pairs for the efficient detection of BSI-related bacteria and Candida species, as well as genes related to resistance to methicillin (mecA), vancomycin (vanA and vanB) and carbapenems (KPC). Primer sequences, gene targets and PCR cycling configuration have been described elsewhere in detail [24]. PCR products were then desalted by binding to magnetic microparticles, washing and elution and lastly analysed through ESI-MS in automated system. The base compositions of detected amplicon strands were calculated from the measured masses and compared with a reference database, which contained the complete amplicon signatures for more than 780 Bacterial and Candida species [24–25]. The BAC BSI assay controls were composed by an extraction control target added to each sample, an internal PCR calibrator in each reaction well and a sterile buffer sample as the negative control in every run [3]. The PCR/ESI-MS results included a report of the organism names, level, and Q score. The level was calculated with reference to the internal calibrator construct, as described previously [30], and allowed a relative approximation of the concentration of any specific target. The Q score ranged between 0 and 1 and it was a relative measure of the quality of the identification. The Q score cut offs were designed to prevent specific identification when the information obtained was not sufficient to confidently resolve an organism’s identity [24]. For this study, a Q score of > 0.90 was considered a reportable result.

### Data analysis

In order to determine the analytical performance of the IRIDICA System all the results obtained with the PCR/ES MS assay were compared with those by standard microbiological testing. A direct comparison between the microorganism isolated in the blood culture and the microorganism detected by IRIDICA was made and matched positive or negative results for each specimen were recorded. When discrepant results between the two methods were found, a constructed “clinical infection criterion” was used to determine whether the discrepant results were clinically significant or not. The clinical records of each individual patient that had discrepant findings were reviewed in order to identify the diagnosed focus of infection, as well as the results of cultures from other specimens available within a time frame of 7 days after and 7 days before the IRIDICA test. McNemar test and Cohen κ were used to determine agreement and concordance in the light of the evaluation of the clinical context.

Common contaminants, such as coagulase-negative staphylococci (CoNS), were considered as potential contaminants for both methods and excluded from the overall analysis, unless more than one set of blood culture was positive for the same organism, which is compatible with a true infection [25, 31].

## Results

The performance of the IRIDICA BAC BSI Assay was evaluated by comparison with the traditional culture-based methods routinely performed in our laboratory.

Of 300 patients enrolled in the study, 10 did not have matching PCR/ESI-MS or standard-of-care microbiology results and were excluded from the final analysis.

In the absence of repeated positivity for known contaminants through multiple sets or a clinical presentation favouring a different conclusion, a single positive result is likely to be the consequence of a contamination event [25]. We therefore excluded from our analysis all the samples positive for the most common contaminants, unless more than one set was positive for the same organism, as described above in the methods section. The specimens excluded for this reason were 29: 7 *Propionibacterium acnes* (24,14%); 7 Fungus not identified (24,14%); 3 *Staphylococcus capitis* (10,34%); 2 *Staphylococcus haemolyticus* (6,9%); 2 *Staphylococcus hominis* (6,9%); 2 *Pseudomonas fluorescens* (6,9%); 1 *Bacillus* spp (3,45%); 1 *Erwinia bilingiae* (3,45%); 1 *Staphylococcus auricularis* (3,45%); 1 S*treptococcus constellatus* (3,45%); 1 S*treptococcus salivarius* (3,45%); 1 *Staphylococcus epidermidis* (3,45%).

**Fig 1:**
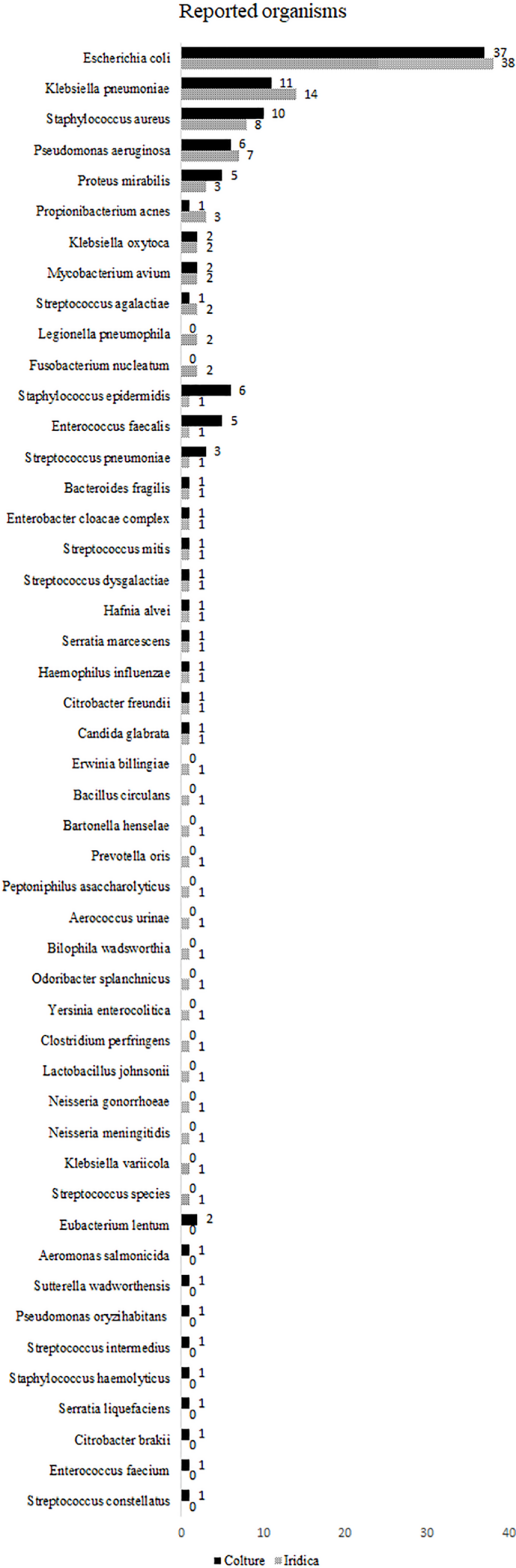
Distribution of the organisms included in the study. The organisms reported by culture (solid bar) and PCR/ESI-MS (patterned bar) are sorted by decreasing order of PCR/ESI-MS reported organisms.

The organisms were detected from BSI with different frequencies that are shown in Fig 1. The distributions of species identified by MALDI-TOF and those detected by PCR/ESI-MS were overall similar.

As shown in Table 1, 222 of the included specimens were concordant, of which 151 yielded negative results. The overall concordance was of 86% with a Cohen κ of 0.68 (95% CI 0,59–0,77). 94 samples presented a positive result for blood culture, of which 71 were in agreement with the molecular assay. The concordance of identification (calculated sensitivity) was therefore of 76% (95% CI, 67–85%), while the calculated assay specificity was of 90% (95% CI 85–95%). Finally, the negative predictive value (NPV) was 87% (95% CI, 82–92%), while the positive predictive value (PPV) was of 82% (95% CI 74–90%).

**Table 1:** Assay performance.

Samples included, *n*	261
Matched negatives, *n*	151
Matched positives, *n*	59
Concordant primary identification with unmatched additional detections, *n*	12
IRIDICA-negative and blood culture-positive detections, *n*	18
IRIDICA-positive and blood culture-negative detections, *n*	16
Unmatching results, *n*	5
Concordance, *%*	86
Sensitivity, *%*	76
Specificity, *%*	90
PPV, *%*	82
NPV, *%*	87

59 specimens were matching at the species level, including 2 specimens showing dual detection. While primary identification was in agreement between the two techniques, a secondary detection was found by PCR/ESI-MS in 6 samples, and by MALDI-TOF in a single instance; in 5 cases, each method had at least a non-matching additional identification (Table 2).

**Table 2:** Concordant primary pathogen identification with unmatched additional detections.

Primary organism(s) detected in concordant PCR/ESI-MS and culture results	Additional detection result by:	
	PCR/ESI-MS	Culture
*Streptococcus spp*.	*Odoribacter splanchnicus*, *Bilophila wadsworthia*	*Sutterella wadsworthensis*
*Citrobacter freundii*	*Klebsiella variicola*	*Citrobacter brakii*
*Escherichia coli*	*Pseudomonas aeruginosa*	*None*
*Hafnia alvei*	*Yersinia enterocolitica*	*Serratia liquefaciens*
*Escherichia coli*	*Clostridium perfringens*	*Klebsiella oxytoca*
*Escherichia coli*	*Klebsiella pneumoniae*	*None*
*Escherichia coli*	*Bacteroides fragilis*	*None*
*Escherichia coli*	*Klebsiella pneumoniae*	*None*
*Pseudomonas aeruginosa*	*Fusobacterium nucleatum*	*Eubacterium lentum*
*Candida glabrata*	*Lactobacillus johnsonii*	*None*
*Enterobacter cloacae*	*None*	*Enterococcus faecalis*
*Klebsiella pneumoniae*	*Fusobacterium nucleatum*	*Enterococcus faecalis*

In 18 samples, blood culture identified an organism that was not reported by PCR/ESI-MS (Table 3). Conversely, PCR/ESI-MS reported a BSI relevant organism in 16 additional specimens that were blood culture negative (Table 4). In 5 cases both methods detected at least one microorganism, but there was no match between the identifications (Table 5).

**Table 3:** IRIDICA-negative and blood culture-positive detections.

Blood culture-reported organism with PCR/ESI-MS negative result	No. of blood culture-positive, PCR/ESI-MS-negative samples	Medical records review
*Staphylococcus epidermidis*	*4*	*Two cases*: *effective antibiotic treatment against Staphylococcus epidermidis/One case*: *clinical data insufficient/One case*: *true bacteremia unlikely*
*Pseudomonas aeruginosa*	*2*	*Urine culture positive for Pseudomonas aeruginosa/Clinical diagnosis*: *sepsis Pseudomonas aeruginosa-related*
*Streptococcus pneumoniae*	*2*	*Detection of Streptococcus pneumoniae urinary antigen/Effective antibiotic treatment against Streptococcus pneumoniae*
*Aeromonas salmonicida*	*1*	*Clinical presentation compatible with Aeromonas spp*. *infection*
*Escherichia coli*	*1*	*Clinical diagnosis*: *sepsis Escherichia coli-related from urinary tract infection*
*Enterococcus faecalis*	*1*	*One additional set of blood culture collected the same day*: *Enterococcus faecalis*
*Streptococcus mitis*	*1*	*True bacteremia unlikely*
*Streptococcus intermedius*	*1*	*Clinical diagnosis*: *sepsis from respiratory tract infection*
*Pseudomonas oryzihabitans*	*1*	*Clinical presentation compatible with Pseudomonas spp*. *infection*
*Klebsiella pneumoniae*	*1*	*Urine culture positive for Klebsiella pneumoniae*
*Enterococcus faecium*	*1*	*Clinical diagnosis*: *sepsis Enteroccocus faecium-related*
*Staphylococcus haemolyticus*	*1*	*Clinical diagnosis*: *sepsis Staphylococcus-related*
*Haemophilus parainfluenzae*	*1*	*Clinical diagnosis of sepsis*

In 18 patients blood cultures identified a potential pathogen which was not detected by IRIDICA. The reliability of these results was achieved reviewing the patients’ clinical data, as well as the results of additional relevant exams from other specimens available.

**Table 4:** IRIDICA-positive and blood culture-negative detections.

PCR/ESI-MS-reported organism with blood culture negative result	No. of PCR/ESI-MS-positive, blood culture-negative samples	Medical records review
*Escherichia coli*	*5*	*Urine culture positive for Escherichia coli/Effective antibiotic treatment against Escherichia coli*
*Klebsiella pneumoniae*	*3*	*Urine culture positive for Klebsiella pneumoniae/Clinical diagnosis of pneumoniae/Klebsiella pneumoniae identified in aortic abscess*
*Pseudomonas aeruginosa*	*2*	*Clinical diagnosis*: *sepsis from urinary tract infection*
*Legionella pneumophila*	*2*	*Detection of Legionella pneumophila urinary antigen*
*Bartonella henselae*	*1*	*Positive serology for Bartonella henselae*
*Neisseria gonorrhoeae*	*1*	*Clinical diagnosis*: *pelviperitonitis Neisseria gonorrhoeae-related*
*Neisseria meningitidis*	*1*	*Clinical data insufficient*
*Streptococcus agalactiae*	*1*	*Clinical presentation compatible with Streptococcus agalactiae infection*

In 16 patients a potential pathogen was detected by the molecular method but not by MALDI-TOF. The review of clinical data and additional relevant exams from other specimens was used for the resolution of discrepancies.

**Table 5:** Unmatching results between the two methods.

Sample ID	Organism(s) identified by PCR/ESI-MS	Organism(s) identified by blood culture test	Relevant additional exams
A.	*Klebsiella oxytoca*	*Proteus mirabilis*	Urine colture result on sample collected the same day: *Proteus mirabilis*
B.	*Bacillus circulans*, *Aerococcus urinae*, *Peptoniphilus asaccharolyticus*	*Proteus mirabilis*, *Enterococcus faecalis*	Four additional sets of blood culture collected the same day: *Proteus mirabilis*, *Enterococcus faecalis*
C.	*Prevotella oris*	*Escherichia coli*, *Bacteroides fragilis*, *Eubacterium lentum*	One additional set of blood colture collected the same day: *Escherichia Coli*
D.	*Haemophilus influenzae*	*Staphylococcus aureus*	One set of blood colture collected the day before: *Staphylococcus aureus*
E.	*Escherichia coli*	*Staphylococcus aureus*	None

In 5 cases IRIDICA and blood culture were positive for different targets. The results of additional relevant exams from other specimens were reviewed in order to identify the diagnosed focus of infection.

## Discussion

Of 300 patients enrolled in the study, we excluded 10 as they did not have corresponding PCR/ESI-MS or standard-of-care microbiology results. As described above, we also excluded from our analysis all samples suggestive of contamination, including fungi detected only by the IRIDICA instrument. All detections of fungi not further identified happened within a short time frame and involved immunocompetent patients whose clinical presentation was not compatible with fungiemia. These detections were therefore considered contaminations during the samples processing. As a further confirmation, none of the patients involved was treated with antimycotics and all of them were discharged with a positive outcome.

Of the included specimens, 222 were in agreement, of which 151 presented concordant negative results, 59 showed perfect concordance for the species identified, and 12 were concordant for primary identification.

When PCR/ESI-MS and MALDI-TOF did not match the identification, further investigation was required regarding the reliability and significance of the data. This was achieved reviewing the patients’ medical records, as well as the results of additional relevant exams from other specimens available within a time window of 14 days. Q score and Level analysis did not prove useful in the resolution of discrepancies: the Q scores of all samples were very high (>96) within the range of reportability, with no significant differences between concordant and discordant results. The Level score, conversely, showed extreme variability even within concordant results, as this is not a quantitative test. It was therefore not possible to draw accurate conclusions based on this parameter.

Of the 18 blood culture-positive, IRIDICA negative specimens (Table 2), clinical data provided strong support to the microbiology result in 15 cases; in 2 cases laboratory and clinical data were not compatible with true bacteremia, while in the remaining case the review of medical records did not provide sufficient information to solve the discrepancy.

Of the 16 PCR/ESI-MS positive, blood culture-negative specimens (Table 3), the review of individual medical record strongly suggested the high likelihood of bacteremia in 14 cases, whereas in 1 sample, while plausible, clinical data were insufficient to reach a definite diagnosis of sepsis. In the remaining case, IRIDICA detected *N*. *meningitidis*, a pathogen which presents potentially severe clinical consequence. In addition to two negative sets of blood cultures, however, laboratory and clinical data were not compatible with meningococcemia, and the patient was discharged without being treated for it. This makes an aspecific reaction during the PCR more likely compared to true bacteremia.

In 5 patients, PCR/ESI-MS and blood culture showed a detection of un-matching organisms (Table 4). In 4 of those cases, clinical data provided strong support to the microbiology result; in the remaining sample, while a true bacteremia was strongly suggested, the medical record did not provide sufficient information to determine the actual causative pathogen. Only the former 4 samples were therefore included in the concordance and discordance calculations.Previous studies show a substantial heterogeneity regarding the performance of this test [3, 25, 30, 32], with a review published in 2016 estimating a sensitivity of 81% and a specificity of 84% [33]. However, all of these studies support our conclusion that the IRIDICA BAC BSI Assay has the ability to identify a broad range of bacteria directly from primary whole blood samples, within eight hours. This might allow a timely administration of a suitable treatment.

These results should nonetheless be confirmed in studies that can directly determine the impact of this approach on clinical and economic outcomes, but also on resistance patterns.

The molecular assay presents some limitations. The provision of results to the clinician is conditioned by several variables, such as the lack of random access to the instrument, which is able to process up to 6 samples in a single batch. Additionally, the presence of an operator is needed at several points during the analysis, amounting to a total hands-on time of approximately 1 h; also, a trained biologist is required to validate the end results. All these conditions make scheduled runs a necessity, outside of which the provision of an end result might be severely delayed, due to the variability in the time window between the collection of the sample and its processability. In addition, as the number of blood cultures drawn per day can exceed 100 in some hospitals, this platform is not suitable for all patients, requiring a strict selection on the clinicians’ part. Furthermore, PCR/ES-MS is unable to provide detailed antimicrobial susceptibility information, unlike culture techniques, and is limited in terms of detecting resistance-associated genes to *mecA*, *vanA*, *vanB*, and KPC. More importantly, the false negative rate is too high to consider this assay suitable as a stand-alone test. For all these reasons, the IRIDICA BAC BSI Assay could be considered a valuable tool if associated with the diagnostic algorithm, but not a replacement for conventional methods.

Also, the PCR/ESI-MS and blood culture are sensitive both to BSI-related pathogens and accidental contaminants introduced into the sample workflow. Sterile laboratory handling and a rigorous technique during blood draws are therefore essential, along with the use of negative controls in the molecular assay. The IRIDICA BC BSI Assay results should therefore be interpreted within the clinical context of the patient and additional laboratory results, similarly to the current microbiology testing. In order to use this assay in routine, a better calibration of the reportability cut-off is needed, considering the high false negative rate. The possibility of random access to the instrument and the capability to process more than 6 samples simultaneously would also be useful improvements.

Additional studies would be required to evaluate the impact on the turnaround time and the consequent clinical management for this laboratory technology.
